# Legal liability of physicians and new governance in the AI era

**DOI:** 10.1007/s11604-026-01997-5

**Published:** 2026-04-29

**Authors:** Daiju Ueda, Takafumi Ochiai, Taichi Kakinuma, Hirokazu Yamaguchi, Akihiro Fukuda, Kenichi Saito, Hirotaka Takita, Yasuhito Mitsuyama, Shannon Walston, Yukio Miki

**Affiliations:** 1 Department of Artificial Intelligence, Graduate School of Medicine, Osaka Metropolitan University, Osaka, Japan; 2 Atsumi & Sakai, Policy Research Institute, Tokyo, Japan; 3 Smart Governance Inc., Tokyo, Japan; 4 STORIA Law Office, Kobe, Japan; 5 LPIXEL Inc., Tokyo, Japan; 6 Department of Diagnostic and Interventional Radiology, Graduate School of Medicine, Osaka Metropolitan University, Osaka, Japan

**Keywords:** Agile governance, Legal liability, Physician liability, Artificial intelligence, Healthcare, Medicine

## Abstract

The rapid integration of artificial intelligence (AI) into clinical practice presents novel legal and ethical challenges that traditional legal frameworks—predicated on human autonomy and predictability—struggle to address. This paper examines the evolving legal liability of physicians and the necessity for new governance models in the era of medical AI. We argue that conventional, static regulatory approaches are insufficient for the fast-evolving nature of AI technologies. Instead, we advocate for “Agile Governance,” a dynamic framework involving continuous rule evaluation by multiple stakeholders, including government bodies, corporations, academic societies, and patient/public representatives. Through the analysis of hypothetical malpractice scenarios, we explore key legal issues: the physician’s role as the ultimate decision-maker, the duty of explaining AI usage, and the emerging responsibilities of AI vendors. Our analysis highlights that liability is increasingly dispersed among physicians, medical institutions, and vendors, complicating the attribution of responsibility. Drawing parallels with the autonomous driving sector, we propose a systemic approach to safety and liability. This includes establishing clear performance standards, creating robust accident investigation mechanisms to facilitate information sharing, and designing collective compensation systems for victim relief. We conclude that physicians must actively participate in designing these social systems to maximize the benefits of AI while appropriately managing the associated risks.

**Supplementary Information:**

The online version contains supplementary material available at 10.1007/s11604-026-01997-5.

## Introduction

Artificial intelligence (AI) technologies are rapidly permeating all areas of medicine, beginning with diagnostic imaging, and are gradually reshaping the daily workflows of physicians [1, 2]. While the rigorous verification of safety and efficacy required in healthcare has tempered the pace of AI implementation into medical practice compared to other fields, the potential benefits are steadily expanding [3]. As we approach this threshold, the intervention of AI introduces legal and ethical challenges not previously anticipated [4]. Specifically, the methodology of clinical decision-making and the attribution of liability in the event of diagnostic errors or unforeseen outcomes represent urgent issues for physicians [5]. This paper examines these complex issues from a legal perspective to provide a foundation for future discussion on how physicians, including radiologists, should navigate this new era [6].

### Limitations of traditional legal systems and the necessity of “Agile Governance”

The legal systems and concepts of liability underpinning our society are largely constructed on the premise of a “rational, autonomous individual” capable of predicting consequences and controlling their actions based on free will [7]. This traditional premise is challenged by the emergence of AI, which operates probabilistically and statistically, often via decision-making processes that are not fully transparent (the so-called “black box problem”) [5, 8].

AI technology evolves at an extremely rapid pace, and its societal impact changes dynamically. Consequently, the conventional approach of establishing fixed rules over several years risks obsolescence by the time such rules are enacted [9]. Furthermore, AI development transcends national borders, and technical information regarding algorithms and training data tends to be unevenly distributed with developers retaining most of the information. Thus, simply applying traditional negligence liability theories could lead to polarized outcomes: either excessively stifling innovation or failing to assign responsibility, thereby compromising safety [9].

This challenge is not unique to AI; earlier general-purpose technologies such as automobiles and the internet also required iterative adjustments in safety regulation, professional standards, and liability allocation. Medical AI, however, intensifies these pressures because its performance may vary across settings, relevant technical information is unevenly distributed, and its outputs shape value-laden clinical decisions.

“Agile Governance” has been proposed as a new framework to address these challenges [10, 11]. This approach avoids fixing rules permanently; instead, it involves continuous evaluation and revision of rules by diverse stakeholders—including governments, corporations, academic societies, patient organizations, and citizen/public representatives—in response to technological and societal progress [12, 13]. Establishing a multi-stakeholder governance structure that combines laws with guidelines, standards, and certifications to respond flexibly to change is essential for the future AI-incorporating society. The practical significance of this governance problem becomes clearer when translated into a concrete malpractice scenario.

### Current legal interpretations and liability structures regarding medical AI

To understand these theoretical challenges concretely, we examine the relationship between physician and AI liability through a hypothetical medical malpractice scenario involving an AI diagnosis support system.

#### Hypothetical scenario

In clinical settings utilizing AI diagnosis support systems, discrepancies may arise between a physician’s clinical judgment and the AI’s output. For instance, a physician may suspect an abnormality based on imaging findings while the AI indicates “no abnormality,” or vice versa. If a physician retracts their own finding to align with the AI, resulting in patient harm, how is legal liability determined? [14, 15].

This issue encompasses three primary legal points.

1. Responsibilities regarding medical practice

As indicated in the 2018 notification from the Ministry of Health, Labour and Welfare, the Medical Practitioners’ Act defines AI strictly as a “tool” to assist physicians; the physician bears the final diagnostic responsibility [16]. This policy choice plausibly reflected the early stage of clinical deployment, serving to preserve existing duty-of-care doctrines while deferring detailed, technology-specific liability rules. Since then, however, AI technologies have become more tightly integrated into clinical workflows, exerting a greater influence on triage and decision support. Accordingly, the continued adequacy of a simple ‘tool’ framing should be reassessed as AI becomes more autonomous and clinically consequential. When contradictions arise between AI and physician findings, the physician should not be expected to rely blindly on the AI but to make a subjective conclusion based on their own professional expertise.

Medical institutions also risk vicarious liability under the Civil Code if a physician employed at their facility uses an AI tool and this leads to patient harm [17].

Crucially, the use of advanced AI does not diminish a physician’s duty of care; rather, it can recalibrate the expected standard of care to include (a) maintaining baseline diagnostic competence independent of AI and (b) exercising competent oversight of AI outputs (e.g., judicious application, adherence to model constraints, and evaluation of discordant outputs) [18].

2. Duty to inform patients

Whether there is a duty to explain the role of AI in the diagnostic process to the patient (i.e., whether it falls under informed consent) is not currently established as a clear legal obligation [19, 20]. This reflects the current positioning of AI as a supplementary tool, with the physician bearing ultimate responsibility. However, if AI assumes a more central role in the future, substantially influencing diagnosis and treatment policies, explanations regarding AI usage may become required as part of informed consent.

3. Liability of AI vendors

The legal liability of vendors providing AI diagnosis support systems is another significant point. Currently, standalone software provided via SaaS (Software as a Service) is generally interpreted as not falling under the definition of a “product” within the Product Liability (PL) Act in Japan [21].

To place these issues in a broader context, several jurisdictions are actively refining regulatory and liability frameworks relevant to AI vendors. For example, the European Union’s Artificial Intelligence Act (Regulation (EU) 2024/1689) adopts a risk-based compliance regime for AI systems, and the updated EU Product Liability Directive (Directive (EU) 2024/2853) explicitly extends strict-liability concepts to software, which may affect AI-enabled medical products. In the United States, the Food and Drug Administration has issued draft guidance addressing lifecycle management and marketing submission expectations for AI-enabled device software. Although these regulations differ in scope and legal theory, they underscore a global trend toward more explicit vendor obligations, including documentation, monitoring, and post-market accountability.

However, this does not imply total exemption for vendors. AI-enhanced physical products likely are covered by the Product Liability Act. Under contract law and general tort law, vendors likely hold a duty of care to provide specific and sufficient explanations or warnings to medical practitioners regarding not only the performance but also the limitations and risks of their systems [7]. Merely stating in the terms of use that “the final judgment is made by the physician” may be insufficient. More proactive support, such as protocols for discrepancies between physician and AI findings and specific information regarding system accuracy, may be required.

#### Dispersion of responsibility

These interpretations highlight the reality that liability involving medical AI is dispersed among multiple entities: physicians, medical institutions, and vendors. For a single adverse event, multiple parties may bear responsibility based on different legal grounds, potentially resulting in joint and several liability toward the patient [22]. Once liability is understood as distributed across multiple actors, the problem shifts from ex post blame allocation to ex ante system design. While this complexity makes simple attribution of blame difficult, it also presents an opportunity for diverse stakeholders to collaborate on improving system-wide safety [23].

### System design lessons from autonomous driving

The challenges facing the medical field share commonalities with the autonomous driving sector, which also aims for the implementation of advanced AI. In autonomous driving, legal and policy discussions are more advanced, particularly with respect to conditional and eventually fully autonomous operation [24–27].

These discussions focus on designing legal systems to improve safety for society as a whole, rather than solely on identifying liable parties. Specific measures include:

- *Setting Clear Standards*: Clarifying and quantifying safety standards and guidelines regarding performance to enhance predictability. This delineates the evaluation metrics, test conditions, and recording obligations that operators must observe, thereby reducing arbitrary judgments in individual cases.
- *Accident Investigation and Information Sharing*: Establishing mechanisms to collect and analyze data regarding accidents and “near-miss” incidents to continuously improve rules and systems. This includes developing accident investigation institutions with specialized expertise. These measures promote societal information sharing to enhance safety and ensure that judgments in accident cases are based on scientific findings. Furthermore, future measures may incorporate obligations to cooperate with investigations and sanctions for non-compliance, shifting the purpose of sanctions from punishing individual failures to encouraging cooperation in cause analysis and recurrence prevention.
- *Establishment of Victim Relief Systems*: To ensure victim relief even when the locus of responsibility is unclear, efforts are underway to clarify interpretations of the compulsory automobile liability insurance system, and collective compensation systems (such as funds or mandatory insurance) are under consideration [28]. These systems envision no-fault, rapid benefits that do not require proof of negligence, with standardized coverage and procedures, followed by subrogation or apportionment among related parties.

These initiatives aim to design a “system” that manages risk and allows society to safely enjoy the benefits of technology, rather than merely questioning the responsibility of individual drivers or manufacturers. This perspective will likely serve as a reference for medical AI in the future.

At the same time, the analogy has limitations. Autonomous driving primarily addresses physical environments with comparatively well-defined sensing and control variables, whereas medicine involves substantial biological heterogeneity, evolving disease processes, individual patient values, and ethically salient trade-offs. Therefore, governance mechanisms in medicine must explicitly incorporate clinical context, patient-centered outcomes, and professional judgment when translating lessons from transportation. The remaining task, therefore, is to translate these general lessons into an institutional architecture suited specifically to medicine.

### Implications for physicians and future perspectives

Based on the foregoing discussion, the following are key points physicians must address moving forward.

#### Integrated system design: physicians, vendors, societies, government, and patients

Relying solely on the duty of care of individual physicians or the duty of explanation of vendors cannot resolve all issues surrounding medical AI. As recent legal discussions and mock trials suggest [14, 15], responsibility tends to be dispersed among multiple entities [7, 9].

The priority should be to improve the overall system and protect patients. To this end, referencing the autonomous driving model, we must build a social consensus by discussing the following three pillars:

1. *Continuous Guideline Development*: Academic societies, in collaboration with regulators, vendors, and patient/public representatives, should proactively and continuously update practical guidelines. These updates should clarify the roles (duties of care) of each stakeholder while protecting reasonable new initiatives.
2. *Construction of Accident Investigation Systems*: Objective information collection and sharing systems should be established for when adverse events occur. This facilitates the sharing of scientific data and aims to establish legal judgments grounded in science and technology.
3. *Establishment of Compensation and Relief Systems*: Apart from personal liability, collective compensation systems (insurance or funds) should be established for rapid victim relief. The scope of events covered, benefit items/limits, timelines, funding distribution, and subrogation rules must be predetermined.

Within such a system, the physician’s duty of care does not disappear; rather, it is recalibrated in light of AI-assisted practice.

#### Shifting “standard of care” and the duty to use AI

As AI performance improves and its use becomes generalized, “diagnosing with AI” may eventually be regarded as the new standard of medical care [7]. In such an era, the failure to utilize high-performance AI could itself be considered a breach of the duty of care [29, 30].

#### Human intervention in high-performance AI

If AI diagnostic accuracy eventually surpasses human capability in certain domains [31], can experts justifiably overturn an AI’s judgment? If a physician overrides an AI result resulting in patient harm, the liability could be significant. Conversely, experienced medical professionals should not blindly follow AI [7]. Academic societies must leverage their expertise to lead the development of guidelines regarding when human intervention in AI judgment is justified [9].

## Conclusion

As this paper has shown, the introduction of medical AI makes responsibility more dispersed, renders static legal rules less adequate, and requires a governance framework combining standards, investigation, and compensation.

AI has the potential to be a powerful partner that extends the capabilities of physicians. Yet its adoption is not merely the addition of a new tool; it requires us to reconsider how responsibility in medicine should be allocated and governed.

As radiologists standing at the forefront of AI technology, we cannot turn away from this discussion [3, 32]. We have a duty to participate actively in the dialogue alongside legal scholars, administrators, vendors, and patients to construct new rules and social systems that maximize technological benefits while appropriately managing risks. It is our hope that this paper serves as a first step toward that goal.

## Supplementary Information

Below is the link to the electronic supplementary material.

Supplementary Material 1
