# Supplementary material for: Legal liability of physicians and new governance in the AI era
Source: Jpn J Radiol. 2026 Apr 29;44(8):1316–20. doi: 10.1007/s11604-026-01997-5 (PMC13400459; doi:10.1007/s11604-026-01997-5)
Supplement: Supplementary file 1 — Supplementary Material 1 [file 11604_2026_1997_MOESM1_ESM.pdf]

AI時代における医師の法的責任と新たなガバナンスの探求

植田大樹[1]，落合孝文[2,3]，柿沼太一[4]，山口宏和[4]，福田明広[5]，
齊藤健一[1]，田北大昂[6]，光山容仁[1,6]，Shannon Walston[1]，三木幸雄[6]

[1] 大阪公立大学大学院医学研究科 人工知能学
[2] 渥美坂井法律事務所・外国法共同事業 / プロトタイプ政策研究所
[3] スマートガバナンス株式会社
[4] STORIA法律事務所
[5] エルピクセル株式会社
[6] 大阪公立大学大学院医学研究科 放射線診断学・IVR学

要旨

人工知能（AI）の臨床現場への急速な統合は、人間の自律性と予測可能性を前提として構築されてきた従来の法的枠組みでは十分に対処しきれない、新たな法的・倫理的課題を提起している。本稿は、医療AI時代における医師の法的責任の変容と、新たなガバナンスモデルの必要性を検討するものである。我々は、急速に進化するAI技術に対して、固定的な規制アプローチでは不十分であると論じ、政府、企業、学会、患者・市民代表など複数の主体が継続的にルールを評価・見直す「アジャイル・ガバナンス」を提唱する。仮想的な医療過誤事例の分析を通じて、医師が最終的な意思決定者であること、AI利用に関する説明義務、AIベンダーに生じつつある責任といった主要な法的論点を検討した。その結果、責任は医師、医療機関、ベンダーの間に分散しつつあり、責任帰属は一層複雑化していることが示された。さらに、自動運転分野との比較を通じて、明確な性能基準の策定、事故調査と情報共有の制度化、被害者救済のための集団的補償制度の設計といった、システム全体を視野に入れた安全・責任設計の必要性を提案する。結論として、医師はAIの便益を最大化しつつ、そのリスクを適切に管理するための社会制度設計に主体的に参画すべきである。

はじめに

人工知能（AI）技術は、画像診断をはじめとする医療のあらゆる領域に急速に浸透し、医師の日常診療のワークフローを徐々に変化させつつある。[1,2] 医療分野では安全性と有効性について厳格な検証が求められるため、AIの社会実装の速度は他分野より抑制されてきたが、その便益は着実に拡大している。[3] こうした転換点に差しかかる中で、AIの介在は、従来は十分に想定されてこなかった法的・倫理的課題を顕在化させている。[4]

とりわけ、診療上の意思決定がいかなる方法で行われるべきか、そして診断エラーや予期せぬ転帰が生じた場合に責任をいかに帰属させるかは、臨床医にとって喫緊の問題である。[5] 本稿は、放射線科医を含む臨床医がこの新たな時代にどのように向き合うべきかを考えるための基盤を提供することを目的として、これらの複雑な問題を法的観点から検討する。[6]

伝統的法制度の限界と「アジャイル・ガバナンス」の必要性

我々の社会を支える法制度や責任概念の多くは、「理性的で自律した個人が、自らの自由意思に基づいて結果を予測し、自身の行為を統制できる」という人間像を前提として構築されてきた。[7] しかし、確率論的・統計的に作動し、その意思決定過程が必ずしも十分に透明ではないAIの登場は、この前提を大きく揺るがしている。[5,8]

AI技術は極めて速く進化し、その社会的影響も動的に変化する。そのため、数年単位で固定的なルールを整備する従来型の手法では、ルールが制定された時点ですでに陳腐化している危険がある。[9] さらに、AI開発は国境を越えて進み、アルゴリズムや学習データに関する技術情報は、しばしば開発者側に偏在する。こうした状況で従来の過失責任論を単純に適用すると、イノベーションを過度に萎縮させるか、あるいは責任の空白を生じさせて安全性を損なうかという、両極端の結果を招き得る。[9]

もっとも、この問題はAIに固有のものではない。自動車やインターネットのような過去の汎用技術もまた、安全規制、専門職基準、責任配分について反復的な調整を必要としてきた。医療AIは、性能が導入環境によって変動し得ること、関連する技術情報が偏在していること、そしてその出力が価値判断を伴う臨床意思決定に直接影響することから、こうした圧力をより顕在化させる。

このような課題に対する新たな枠組みとして、「アジャイル・ガバナンス」が提唱されている。[10,11] これは、一度定めたルールを固定化するのではなく、政府、企業、学会、患者団体、市民・公共の代表といった多様なステークホルダーが、技術や社会の変化に応じて継続的にルールを評価・見直していく考え方である。[12,13] 法律のみならず、ガイドライン、標準、認証などを組み合わせたマルチステークホルダー型のガバナンスを構築し、変化に柔軟に対応することが、AIを組み込んだ将来社会では不可欠となる。このガバナンス上の課題の実質的な意味は、具体的な医療過誤場面に置き換えることでより明確になる。

医療AIをめぐる現在の法的解釈と責任構造

仮想事例

AI診断支援システムが導入された臨床現場では、医師の臨床判断とAIの出力が一致しない場面が生じ得る。たとえば、医師は画像所見から異常を疑っているにもかかわらず、AIは「異常なし」と判定する場合や、その逆の場合である。もし医師がAIの結果に合わせて自身の所見を撤回し、その結果として患者に不利益が生じたとき、法的責任はどのように判断されるのだろうか。[14,15]この問いは、主として三つの法的論点を含んでいる。

1. 医療行為に関する責任

2018年の厚生労働省通知が示すとおり、現時点の医師法上の整理では、AIはあくまで医師の診断を補助する「道具」であり、最終的な診断責任は医師が負うとされている。[16] この整理は、臨床導入がまだ初期段階にあった当時、既存の注意義務法理を維持しつつ、AI固有の責任ルールの詳細化を将来に委ねるという政策判断を反映したものと理解し得る。もっとも、その後AIはトリアージや意思決定支援を含む臨床ワークフローの内部により深く組み込まれ、その影響力を増している。したがって、AIがより自律的かつ臨床的に重要な役割を担うようになるにつれ、単純な「道具」という把握の妥当性は改めて検討されるべきである。医師の判断とAIの出力が矛盾する場合であっても、医師はAIに盲従するのではなく、自らの専門的知見に基づいて主体的に結論を導かなければならない。

また、雇用されている医師がAIツールを使用した結果として患者に損害が生じた場合には、医療機関自体も民法上の使用者責任を問われる可能性がある。[17]

重要なのは、高度なAIを用いること自体が医師の注意義務を軽減するわけではないという点である。むしろ、AIの導入は期待される注意義務の内容を再調整し、少なくとも、(a) AIに依存しない基礎的な診断能力を維持すること、(b) AIの出力に対して適切な監督を行うこと——具体的には、適切な場面での利用、モデルの制約の遵守、医師判断とAI出力が食い違う場合の吟味——を含む方向へと変化させうる。[18]

2. 患者への説明義務

診断過程におけるAIの役割について患者に説明する義務、すなわちインフォームド・コンセントの対象となるかどうかは、現時点では明確な法的義務としては確立していない。[19,20] これは、現在の法的整理においてAIが補助的ツールにとどまり、最終責任は医師が負うとされていることを反映している。

しかし将来的に、AIが診断や治療方針の形成に実質的かつ中心的な影響を及ぼすようになれば、AI利用の有無やその役割についての説明が、インフォームド・コンセントの一部として求められる可能性が高まるだろう。

3. AIベンダーの責任

AI診断支援システムを提供するベンダーの法的責任も、重要な論点である。日本では、SaaS（Software as a Service）として提供されるソフトウェア単体は、一般に製造物責任法上の「製造物」には該当しないと解されている。[21]

しかし、このことはベンダーが全面的に免責されることを意味しない。まず、AIを組み込んだ有体物については、製造物責任法の適用対象となる可能性が高い。さらに、契約法上および一般不法行為法上、ベンダーには、製品の性能のみならず、その限界やリスクについて、医療従事者に対して具体的かつ十分な説明・警告を行う注意義務が認められ得る。[7] 利用規約に「最終判断は医師が行う」と記載するだけでは不十分であり、医師の所見とAIの判断が不一致となった場合の対応手順や、精度・適用範囲・限界に関する具体的情報を含む、より積極的な支援が求められる可能性がある。

これをより広い文脈で見ると、AIベンダーを取り巻く規制・責任枠組みは海外でも明確化が進んでいる。たとえば欧州連合では、AIシステムに対するリスクベースの適合義務を定めるAI法（Regulation（EU）2024/1689）や、ソフトウェアを厳格責任の対象に明示的に含める改正製造物責任指令（Directive（EU）2024/2853）が整備されつつあり、AI搭載医療製品にも影響を及ぼし得る。米国でも、FDAがAI対応医療機器ソフトウェアに関するライフサイクル管理や承認申請に関するドラフトガイダンスを公表している。各制度の射程や法理は異なるものの、文書化、モニタリング、市販後の説明責任を含むベンダー義務をより明示化する方向への国際的潮流がうかがえる。

責任の分散

以上の解釈から明らかになるのは、医療AIに関する責任が、医師、医療機関、ベンダーという複数の主体に分散しているという現実である。単一の有害事象について、異なる法的根拠に基づき複数当事者が患者に対して責任を負い、場合によっては連帯責任に近い構造をとる可能性もある。[22] この複雑さは、単純な責任帰属を難しくする一方で、システム全体の安全性向上に向けて多様な主体が協働する契機ともなり得る。[23]

自動運転に学ぶ制度設計

医療分野が直面している課題は、高度なAIの社会実装を進める自動運転分野と重要な共通性を持つ。自動運転の領域では、条件付き、さらには完全自動運転を視野に入れて、法制度や政策の議論が先行して進められてきた。[24-27]

そこでは、個別の責任主体を確定することだけに重点を置くのではなく、社会全体の安全性を向上させるための法制度の設計が目指されている。具体的には、次のような取り組みがある。

・明確な基準の設定

性能に関する安全基準やガイドラインを具体化・定量化し、予見可能性を高める。これにより、事業者が遵守すべき評価指標、試験条件、記録義務などが事前に可視化され、個別事案ごとの恣意的な判断を抑制できる。

・事故調査と情報共有

事故やヒヤリハットに関する情報を収集・分析し、ルールやシステムを継続的に改善する仕組みを整備する。加えて、専門的知見を有する事故調査機関を設けることにより、事故対応における判断が科学的知見に立脚したものとなる。さらに将来的には、調査への協力義務や、その不履行に対する制裁を制度化する可能性もある。ここでは、制裁の目的が個別の失敗を罰することから、原因究明と再発防止への協力を促すことへと転換していく。

・被害者救済制度の確立

責任の所在が直ちに明らかにならない場合であっても被害者を救済できるよう、強制保険や基金を含む集団的補償制度が検討されている。[28] そこでは、過失の立証を前提としない無過失・迅速給付を基本とし、給付範囲や手続を標準化したうえで、支払後に保険者等が関係主体間で求償・按分を行う設計が想定されている。

もっとも、この比較には限界もある。自動運転が主として物理環境におけるセンシングと制御の問題を扱うのに対し、医療は生物学的な異質性、変化し続ける病態、個々の患者の価値観、そして倫理的に重要なトレードオフを含む。したがって、交通分野からの教訓を医療へ移植する際には、臨床文脈、患者中心のアウトカム、専門職としての判断を制度設計の中に明示的に組み込まなければならない。残された課題は、これら一般的教訓を、医療に適した制度アーキテクチャへと翻訳することである。

医師への示唆と今後の展望

統合的な制度設計：医師・ベンダー・学会・政府・患者

医療AIをめぐる課題は、個々の医師の注意義務や、ベンダーの説明義務だけでは解決できない。近年の法的議論や模擬裁判の検討でも、責任が複数主体へ分散していく傾向が示されている。[7,9,14,15]

重要なのは、患者保護を中心に据えつつ、システム全体を改善することである。そのためには、自動運転分野の知見も参照しながら、少なくとも以下の三つの柱について社会的合意を形成していく必要がある。

1. 継続的なガイドライン整備

学会は、規制当局、ベンダー、患者・市民代表と協働し、実践的なガイドラインを継続的に更新すべきである。これにより、合理的な新規取り組みを保護しつつ、各ステークホルダーの役割と注意義務を明確化できる。

2. 事故調査制度の構築

有害事象が生じた際の客観的な情報収集・共有制度を整備し、科学技術に立脚した法的判断を支える基盤を構築する必要がある。

3. 補償・救済制度の確立

個人責任の追及とは別に、保険や基金による集団的補償制度を整備し、迅速な被害救済を可能にすべきである。対象事象、給付項目・上限、申請から給付までの期間、財源分担、支払後の求償ルールなどをあらかじめ定めておく必要がある。

変容する医療水準とAI利用義務

AIの性能が向上し、その利用が一般化すれば、「AIを用いて診断すること」自体が新たな医療水準とみなされる可能性がある。[7] その段階では、高性能なAIを利用しなかったこと自体が注意義務違反と評価される場面も想定される。[29,30]

高性能AIへの人間の介入

将来的に、特定領域でAIの診断精度が人間を上回るようになった場合、専門家はいつ、どのような根拠でAIの判断を覆すことができるのだろうか。[31] 医師がAIの結果を覆したことで患者に不利益が生じた場合、その責任は重く問われ得る。一方で、経験ある医療専門職がAIに無批判に追従することも許されない。[7] したがって、どのような場合に人間の介入が正当化されるかについては、学会が専門性を活かしてガイドライン整備を主導すべきである。[9]

結論

医療AIの導入は、責任をより分散させ、固定的な法的ルールの妥当性を相対化し、基準、調査、補償を組み合わせた新たなガバナンス枠組みを必要とする。

AIは医師の能力を拡張する強力なパートナーとなり得る。しかし、その導入は単なる新しいツールの追加ではなく、医療における責任をいかに配分し、いかに統治するかを再考する契機である。

AI技術の最前線に立つ放射線科医を含む医師は、この議論から目を背けることはできない。[3,32] 法学者、行政、ベンダー、患者とともに、技術の恩恵を最大化しつつリスクを適切に管理するための新たなルールと社会システムを構築する対話に、主体的に参画する責務がある。本稿がその一助になれば幸いである。

※参考文献は本文参照
